# On Change of Soil Moisture Distribution With Vegetation Reconstruction in Mu Us Sandy Land of China, With Newly Designed Lysimeter

**DOI:** 10.3389/fpls.2021.609529

**Published:** 2021-02-18

**Authors:** Yiben Cheng, Wenbing Yang, Hongbin Zhan, Qunou Jiang, Mingchang Shi, Yunqi Wang, Xinle Li, Zhiming Xin

**Affiliations:** ^1^School of Soil and Water Conservation, Beijing Forestry University, Beijing, China; ^2^Jinyun Forest Ecosystem Research Station, School of Soil and Water Conservation, Beijing Forestry University, Beijing, China; ^3^Institute of Desertification, Chinese Academy of Forestry, Beijing, China; ^4^Department of Geology and Geophysics, Texas A&M University, College Station, TX, United States; ^5^Inner Mongolia Dengkou Desert Ecosystem National Observation Research Station, The Experimental Center of Desert Forestry, Chinese Academy Forestry, Beijing, China

**Keywords:** rainfed Pinus sylvestris var. Mongolia, infiltration, semi-arid region, vegetation restoration, soil moisture

## Abstract

**Background:**

China’s so-called Three North Shelterbelt Program (3NSP) has produced a vast area of lined forest reconstruction in the semi-arid regions. This study uses the lined rain-fed Pinus sylvestris var. mongolica (PSM) sand-fixing forest in the eastern part of Mu Us Sandy Land in Northwestern China as an example to investigate the ecohydrological process in this region. Rain gauges, newly designed lysimeters and soil moisture sensors are used to monitor precipitation, deep soil recharge (DSR) and soil water content, where DSR specifically refers to recharge that can reach a depth more than 200 cm and eventually replenish the underneath groundwater reservoir.

**Results:**

This study shows that there are two obvious moisture recharge processes in an annual base for the PSM forest soil: a snowmelt-related recharge process in the spring and a precipitation-related recharge process in the summer. The recharge depth of the first process can reach 180 cm without DSR occurring (in 2018). The second process results in noticeable DSR in 2018. Specifically, the DSR values over 2016–2018 are 1, 0.2, and 1.2 mm, respectively. To reach the recharge depths of 20, 40, 80, 120, 160, and 200 cm, the required precipitation intensities have to be 2.6, 3.2, 3.4, 8.2, 8.2, and 13.2 mm/d, respectively. The annual evapotranspiration in the PSM forest is 466.94 mm in 2016, 324.60 mm in 2017, and 183.85 mm in 2018.

**Conclusion:**

This study concludes that under the current precipitation conditions (including both dry- and wet-years such as 2016–2018), water consumption of PSM somewhat equals to the precipitation amount, and PSM has evolved over years to regulate its evapotranspiration in response to annual precipitation fluctuations in Mu Us Sandy Land of China.

## Introduction

The process of desertification is one of the major issues of desert scientific research, which directly impacts the survival of human beings in the affected area (Chen et al., 2019). The Mu Us Sandy Land is one of the four major sandy lands in China, and it is a typical intersecting area of agriculture and husbandry in northern China (Chen et al., 2019). The Mu Us Sandy Land ecosystem has been in the process of restoration and degradation, then researches on the desertification process of the Mu Us Sandy Land have important ecological and social significance (Li et al., 2017). Semi-fixed dunes constitute the main aeolian landscape of the Mu Us Sandy Land. Due to human activities such as aggressive land reclamation, desertification in this area has become increasingly serious (Zhao et al., 2020). With the implementation of a series of ecological projects such as China’s Three North Shelterbelt Program (3NSP) and the conversion of farmland to forests, the trend of desertification of the Mu Us Sandy Land has finally begun to reverse (Becerril-Pina et al., 2015; Cheng et al., 2020a). Despite of many studies by various scholars, the cause of desertification reversal in the Mu Us Sandy Land has not been conclusively determined (Lavee et al., 1998). Some scholars generally believe that the process of water redistribution in semi-arid regions has an important impact on the process of desertification (Liu et al., 2020; Strassburg et al., 2020).

Soil moisture is a vital component in the ecological environment and is the source of life for plants on the earth surface (Sharma and Sharma, 2005). On the land surface of the Earth, not only the natural vegetation distribution is limited by water supply, but also the production of artificial vegetation relies on water supply more than any other factors (Gerten et al., 2004; Contreras et al., 2011). Therefore, the dynamic relations among soil, vegetation and soil moisture and the study on their regulation mechanism are of great concern in various disciplines like forestry, agriculture, livestock, and environment (Young, 1989; Li X. et al., 2013). For instance, the research in this field in the past decade has profoundly influenced the economic development strategies of arid and semi-arid regions (Cao et al., 2011), and lessons learnt can be applicable for management of arid and semi-arid regions in other parts of the world as well.

Pinus sylvestris var. mongolica (PSM) is a geographical variant of European red pine in the Far East (Li et al., 2004; Bao, 2015). It is naturally distributed in large areas of China’s humid and semi-humid areas, especially Daxinganling in the northeast. It inherits the original European red pine’s adaptive capability to a variety of ecological environments, and has characteristics such as heliophile, drought-resistant, cold-resistant, soil infertility-resistant and is more resilient to water shortage. It is one of the most common species used in the so-called 3NPS in China (Zhu et al., 2006). PSM has become the preferred afforestation tree species in arid and semi-arid aeolian sand regions in western China due to its characteristics of drought resistance, cold resistance, rapid growth rate, windproof and sand fixation (Hong et al., 2018). Previous studies on PSM forest mainly focused on the construction and management of PSM forest, its decay mechanism and adaptability (Liu et al., 2019). The impact of PSM forest on soil moisture dynamics and DSR is much less studied.

Since the start of 3NSP in 1978, PSM has been introduced and planted on a large scale in the windy and sandy areas in the Northeast, Northwest, and North China (Runnström, 2000) and this is why we choose it as an example to study. Up to present, it has been applied in more than 300 counties across 13 provinces, municipalities and autonomous regions in China, with a total area over 3 × 10^5^hm^2^(Hu et al., 2008). However, even with such an noticeable achievement, there are still grave concerns in managing windbreak and sand-fixation rain-fed forests (Wang et al., 2010; Cao et al., 2011). For example, reforestation is not successful in some places in 3NSP with unclear reasons. Specifically, trees grow into dwarf trees, which are ineffective in battling land degradation, or even die.

In an attempt to understand why the reforestation effect is successful in some regions and unsuccessful in some other regions, researchers carry out extensive investigations. Some previous studies focus on a number of issues such as transpiration of a single plant such as PSM, interrelationship of atmospheric moisture and PSM in arid regions, and physiological mechanism of PSM to utilize water resources under drought stress (Song et al., 2014). PSM changes soil nutrients in various soil layers and regional soil particle sizes after afforestation (Zeng D. et al., 2009). Furthermore, there are evidences that PSM in sandy land not only fixes the sand, but also changes the microclimate of the sandy land (Zhao et al., 2012), including transpiration in main growing season of PSM and its relationship with canopy micrometeorology (Zheng et al., 2012). Seasonal water consumption characteristics of PSM are different (Zeng D. et al., 2009; Song et al., 2016). However, there are relatively few studies on redistribution of precipitation-related regional water resource in soil layers after vegetation restoration using PSM, and infiltration-percolation process in PSM forest in sandy land is also largely unknown. Few studies have been conducted on whether the deep soil recharge (DSR) has changed or not in the sandy land after planting PSM, where the DSR refers specifically to recharge that can reach a depth more than 200 cm below ground surface and may eventually replenish the groundwater reservoir. *In situ* experiments will help us unravel the interrelationships of precipitation, surface water, soil water and groundwater after planting PSM in the Mu Us Sandy Land of China, which will be the objective of this investigation.

To achieve this objective, two central questions have to be answered. First, is there enough precipitation for the reforestation of PSM in battling land degradation in the Mu Us Sandy Land? Second, what is the change of soil moisture dynamics of sand-fixing PSM forest in a semi-arid region? We will try to answer these questions by inspecting the relationship of precipitation, soil moisture change, and DSR. In particular, if DSR can be detected in the PSM forest land, which means that the precipitation not only satisfies the survival of PSM, but also provides excess water resource to replenish deep soil moisture or, groundwater. To answer these questions, this study uses a newly developed DSR lysimeter to monitor and analyze a typical PSM forest in the Mu Us Sandy Land (Cheng et al., 2017). The most advantage of the new lysimeter is to reduce the disturbance of the soil layer above the measurement layer and to avoid the influence of instrument barrier on lateral flow of soil moisture during the observation stage (Cheng et al., 2020b). The conventional lysimeter has many disadvantages in monitoring large trees as mentioned above. One of such disadvantages is that the closed container will affect the normal growth of the PSM root system. The new instrument does not have this problem (Cheng et al., 2020a). Specifically, we try to tackle the following issues: (1) the sources of soil water recharge, especially the source of deep soil layer moisture; (2) the precipitation density that causes infiltration and its maximal penetrating depth; (3) the rate of annual precipitation-induced infiltration; and (4) the evapotranspiration of the PSM forestry. The ultimate goal of the research is to monitor the rain-water redistribution link of PSM forest land, and through years of continuous observation, to discover the rules and characteristics of deep leakage of rain-fed PSM forest land, and to judge whether rain-fed Pinus sylvestris forest can develop sustainably.

## Materials and Methods

### Overview of the Study Area

**FIGURE 1:**
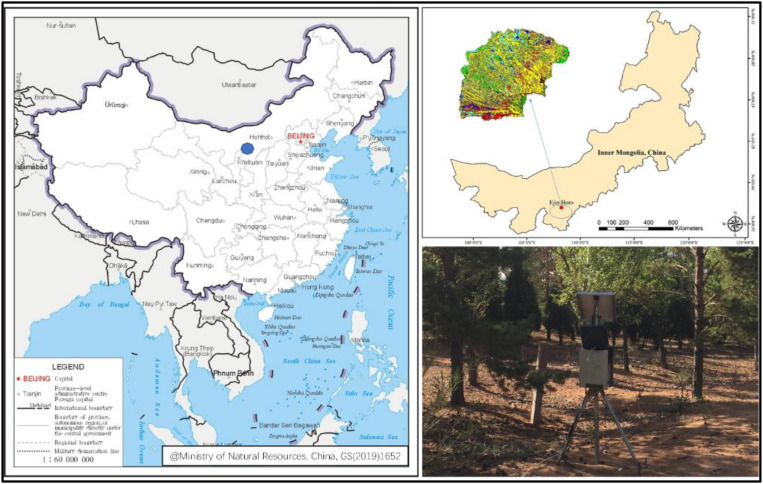
Geographic location of the experimental area and study site.

The study area is located in Chagan Naoer, on the northeastern edge of Mu Us Sandy Land (39°05′16.2″N, 109°36′04″E), as shown in Figure 1. The Mu Us Sandy Land mainly consists of semi-fixed and fixed sand dunes, adjacent with the Loess Plateau, located in a desert-loess transitional zone. It has northwestern wind in the winter with a typically dry winter climate and frequent sandstorms. It has southeastern monsoon in the summer. The summer climate is relatively humid, and it is easy to form local heavy precipitation. The multi-year average precipitation is 400 mm, mostly concentrated during the summer. The groundwater table depth varies between 2 and 17 m in the Mu Us Sandy Land, and it is 8 m at the experimental area of this study (Runnström, 2003). The groundwater table is lower in the summer and higher in the spring, with a variation less than 1.5 m. Since the initiation of 3NSP at the Mu Us Sandy Land in 1989, PSM has been planted in lines in the experimental area, with a 10 m line spacing, an average plant height of 6 m, and an average crown diameter of 6.6 m. The seasonal frozen soil period in the experimental area is from January to March and from November to December in an annual base (Li W. et al., 2013). The soil type in this area is sandy soil and the particle size distribution of 0-200 cm depth is as follows: coarse sand of 3.23%, middle sand of 50.53%, find sand of 36.06%, very fine sand of 7.19%, and silt sand of 2.99%.

### Experimental Plot Design

**FIGURE 2:**
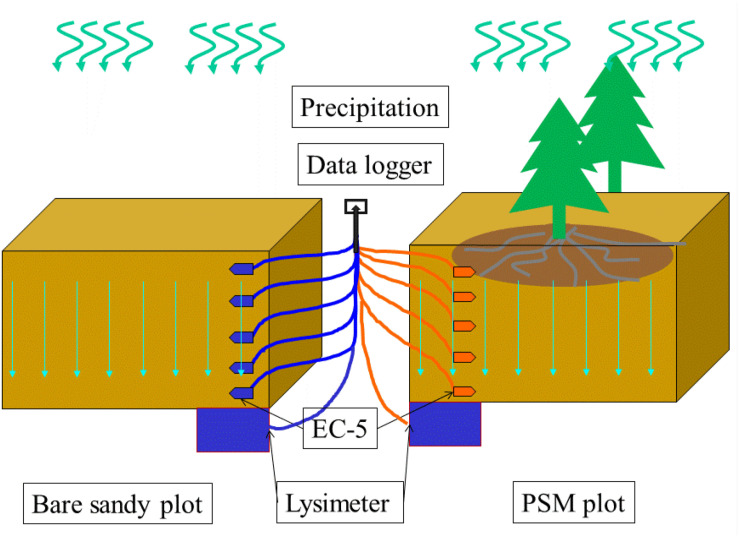
Experimental plot setup.

The experimental design is shown in Figure 2. In order to compare and study the soil moisture changes after the vegetation reconstruction of PSM, this study selects a typical PSM forest in the Mu Us Sandy Land as the research site and selects the adjacent bare sandy land as a comparison study plot. The precipitation, soil moisture, and DSR have been continuously observed over a 3-year period (2016–2018) to answer some of the key questions raised in the introduction of this study.

The canopy of PSM is capable of intercepting precipitation, thus affects the measurements of precipitation and soil moisture underneath the canopy. Therefore, the measurements of this study are made in the middle between the forest lines, measuring precipitation without the interference of the PSM canopy.

When installing the instrument, it is important not to disturb the *in situ* soil layer and the PSM root system. In order not to disturb the original structure of the soil layer, we need to pre-water the test plot before installing the instrument. Watering the test plot can help consolidate the sand layer for excavation because the dry sand is easy to collapse. The first step is to excavate a depth of 3 m soil profile in the middle of two rows of PSM forest with a horizontal cross-section of 0.5 m by 0.5 m. The reason to excavate a 3 m depth soil profile is because the depth of the measured soil layer is 2 m and the height of lysimeter that will be installed below is 1 m. The second step is to install the lysimeter which is 1 m high, 0.5 m wide, and 0.5 m long horizontally. The distance from the top of the lysimeter to the ground surface is 2 m. The third step is to use *in situ* soil to backfill the excavation. During this backfilling process, we also install soil moisture probes above the lysimeter uniformly over the 200 cm soil layer with a depth interval of 40 cm except the shallowest two probes which have a depth interval of 20 cm. One also needs to continuously water the backfilled sands to assist the sand settlement. After the completion of backfill, one needs to wait for 1 year to make sure that the soil layers can be restored to its pre-excavation status as much as possible. As a comparison study, we repeat above procedures and install an identical lysimeter and soil moisture probes in a nearly bare sand plot in exactly the same fashion as in the PSM forest plot. After completing above tasks, one can continuously record and observe the movement of precipitation-induced infiltration in the soil layers of the two test plots. A point to note is that the water table in the site is sufficiently deep (8 m below ground surface) so it will not affect the soil moisture dynamics above the lysimeter, which is exclusively controlled by the precipitation-induced infiltration, root system of PSM, and soil layer structure of the site.

#### Pinus Sylvestris var. Mongolica Root System Excavating

Previous studies in this area have shown that PSM has a shallow root distribution and few underdeveloped taproots, the PSM roots are mostly distributed over a vertical range of 0–2.5 m and rely on precipitation for water supply. The root distribution of PSM can reach 6.5 m horizontally, with a concentrated area of 3 m. The root distribution characteristics of PSM reflects the source of water supply in this area. In general, if the root is distributed horizontally in the shallow soil, it indicates that precipitation is the main water source; if the root is distributed in the deep soil layers, it indicates that the deep soil moisture is the water supply to PSM. In order to accurately investigate the root distribution of PSM in the Mu Us Sandy Land, we have excavated five adjacent PSMs which were planted at the same time as the PSM of concern and use them as surrogates to examine the root distribution layer by layer in a 20 cm depth interval. To avoid root breakage, the sandy soil samples were watered first before being excavated to examine the roots. The roots of each layer were collected and dried and the average biomass of each layer for the five surrogates was calculated and used as the representative biomass of a specified soil layer.

#### Soil Moisture Monitoring

There are many effects of vegetation on soil, including soil nutrient change and biomass change. A most important reason for planting PSM in this area is to fix sand, thus this study focuses on a key factor affecting the survival of PSM in the Mu Us Sandy Land – soil moisture. To fulfill this objective, a soil section is cut out in the middle between two forest lines. The section consists of a layer of dead tree leaves, a leached layer, a depositional layer, and a native soil layer, which is of fine sand. According to the soil stratification of PSM, soil moisture probes (EC-5, accuracy 0.1% mm, United States) have been placed in soil layers at 20, 40, 80, 120, 160, and 200 cm depths. The measurement interval is 1 h. The EC-5 soil moisture probes are used and the correction equation is (Wu et al., 2014):

(1) $$\begin{array}{c} y_{\text{Sandy}}=1.0223\,x_{\text{Sandy}}-0.0302 \end{array}$$

where *x*_*sand*_ and *y*_*sand*_ are the measured value and the corrected soil moisture values, respectively, The root mean square error (RMSE) is used to calculate the total difference between the measured value and the corrected soil moisture values (Wu et al., 2014), and the coefficient of determination for above Eq. 1 is *R*^2^ = 0.9098. The topographic variation of the area is almost negligible and long-term observations show that there is no surface runoff. Under low temperature conditions in the winter, the accuracy of soil moisture probes (EC-5) may drop by 5% (according to the original manufacturer’s instruction). To avoid the possibly unreliable data in the winter, this research focuses on analyzing the data from March to November (unfrozen ground period).

The soil moisture probes clearly showed the actual precipitation-induced infiltration in each soil layer. In this study, six soil moisture probes were installed according to the soil stratification to monitor the infiltration rate and depth after each precipitation event. According to the depth of soil moisture migration, the infiltration depth associated with different precipitation intensities during the observation period can be determined. At the same time, according to the soil water content, the soil water retention in each layer can be calculated as well. Surface evapotranspiration can be calculated according to precipitation, soil water retention, and DSR.

#### DSR Monitoring

The conventional methods to study the process of evapotranspiration focus on the study of the surface evaporation, vegetation transpiration, surface runoff, vegetation interception, etc. In this study, we use a new experimental method to observe the precipitation induced infiltration-penetration process, and directly measure the DSR of the deep soil layer below the root system layer. Through the measurement of water input, soil water storage and deep infiltration as output, the evapotranspiration can be calculated by water balance equation.

**FIGURE 3:**
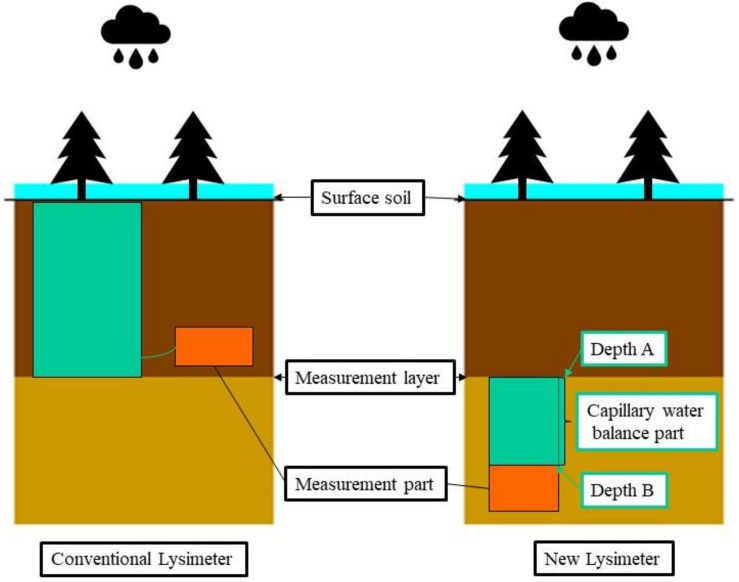
The schematic plot of a new lysimeter (on the right) with respect to the conventional lysimeter (on the left).

To study the soil moisture distribution of PSM in the Mu Us Sandy Land, two sets of data need to be collected: precipitation from a rain gauge, as input of moisture, and DSR measurement from a lysimeter, as output of moisture. The conventional lysimeter is bulky and expensive, the vegetation to be measured have to be planted in a cylindrical container, and the deep soil moisture in the lower part is exported to the measuring part. This new lysimeter has a few innovations (see Figure 3) that can be outlined as follows. The new design has its upper boundary at a designed depth to measure the amount of DSR (denoted as depth-A). A cylindrical container with a diameter of 30 cm with impermeable walls (60 cm) called capillary water balance part is installed from depth-A downward to a deeper depth-B. The length of this part is determined according to the capillary rise of the *in situ* sandy soil, which can be calculated based on soil hydraulic properties. More specifically, the length of AB is greater than the height of capillary rise and it is usually greater than 0.6 m (Liu et al., 2014). At the soil surface there is a rain gauge to measure the amount of precipitation and at the base of the instrument (depth-B), a water collection device is used to measure the amount of water (DSR) exiting the base downward (Cheng et al., 2017).

When installing the instrument, it is necessary to excavate a soil profile on the side of the PSM. Sandy soil is easy to collapse, so we need to water the experimental plot first to make the whole soil layer relatively compact. The length of the instrument is 1.2 m, and the depth of the experimental soil layer is 220 cm, so we need to excavate the soil to a depth of 3.2 m first, then excavate a volume of 0.3 × 0.3 × 01.2 m^3^ (the size of the instrument) laterally to place the instrument. Use the *in situ* soil to back fill, and then continue to water the whole soil layer to make the excavated soil relatively compact. The instrument is made of FRP composite material, which is strong enough to against any cave in problems according to our field experiences. In our previous research, we found that sandy soil can be restored to its original state in less than 3 months, and the instrument contacts well with the soil layer. Before taking the measurements, the lysimeter needs to be placed 1 year in advance, in 2015, allowing the plot soil to reach its pre-installation condition by going through naturally settlement. The annual precipitation in 2015 was 186.4 mm, which is a dry year. In this study, the new instrument is buried at a depth of 2.2 m, below the PSM root layer.

## Results and Discussion

### Annual Characteristics of Precipitation – Soil Moisture Variation

**FIGURE 4:**
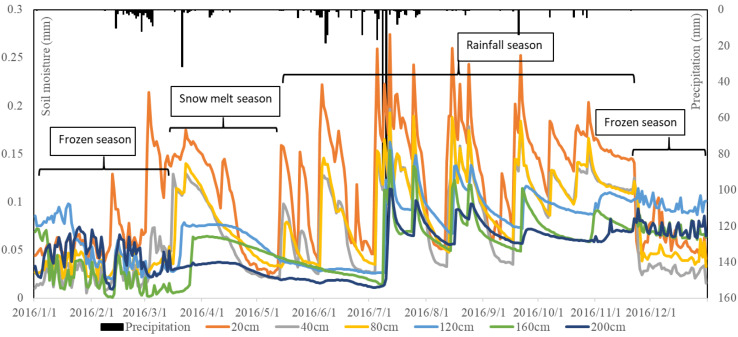
Annual precipitation and soil moisture of each layers in 2016.

The soil moisture variation of PSM in 2016 is shown in Figure 4. It reveals that soil moisture of PSM forest land has obvious seasonal trends. The soil from January to March is frozen, and freezing will cause inaccurate measurement results of shallow soil moisture probes (EC-5), in the following calculation and analysis we selected soil moisture data from March 1 to November 1. The near surface soil moisture recharge is from snowmelt. When the near surface frozen soil starts to thaw, soil at the 20 cm depth is recharged on February 9, 16, and 26 in 2016. Soil at depths greater than 20 cm remains relatively stable. Frequent precipitation events usually occur from June to November, during which soil moisture changes considerably, and soil moistures at different depths exhibit periodic increase or decrease, regulated by the interplay of precipitation and evapotranspiration. After February 26, 2016, soil gradually thaws completely. Figure 4 shows that snowmelt can recharge the soil moisture as deep as 160 cm. The soil moisture at 200 cm depth is recharged for the first time after a heavy precipitation event on July 8, 2016.

According to Figure 4, the soil moisture of the upper 200 cm soil layer fluctuates multiple times in 2016. After November, the soil moisture of the upper 200 cm soil layer fluctuations but DSR is not detected. This is probably due to the error of the EC-5 probe under frozen winter condition. Therefore, the active research period has been revised to a window from March to November each year. Although the precipitation time and intensity are different in the 3-year period of observation (2016–2018), the variation trends of soil moisture in each season of the 3 years are basically the same.

### Root Distribution Characteristics of PSM

Pinus sylvestris var. mongolica in the study site has one taproot and numerous fine roots. The five excavated PSMs have an average taproot length of 4 m, and the vertical distribution of root reflects the water use range of plants. As shown in Table 1, almost all fine roots of PSM are distributed in the shallow soil layer of 0–100 cm depth and the biomass of 0–60 cm root accounts for 85.36% of all the root biomass. This indicates that PSM is effective to utilize soil moisture in shallow layers, but is less effective to utilize soil moisture below 100 cm. Although the main root of PSM has a depth of nearly 4 m, the fine root biomass on the deep taproot (deeper than 100 cm) is very small. Therefore, PSM in this area can be classified as shallow root species. The root distribution also indicates that precipitation is the main water source for PSM in this area. As 90% of the fine roots of PSM are distributed in shallow soil layers (with depths less than 100 cm) in which the water moistures generally fluctuate greatly in a daily and seasonal basis, PSM in the Mu Us Sandy Land is heavily dependent on precipitation events.

**TABLE 1:** Vertical distribution of root biomass of PSM.

Depth (cm)	Sample 1	Sample 2	Sample 2	Sample 4	Sample 5	Average fine root biomass (*d* < 0.2 cm)	Average total root biomass (g)	Biomass percentage of whole layer
0–20	1989.31	1845.89	2000.21	1789.45	1879.45	**1600.86**	**1900.86**	11.14
20–40	5976.45	6120.65	5789.34	6018.83	5729.39	**5646.93**	**5926.93**	34.75
40–60	6783.62	6682.50	6831.72	6391.58	6972.91	**6472.47**	**6732.47**	39.47
60–80	192.83	189.31	194.38	179.37	189.30	**29.04**	**189.04**	1.11
80–100	184.03	198.31	187.30	179.82	179.45	**9.78**	**185.78**	1.09
100–120	168.53	157.82	165.20	177.43.	165.59	**0.1**	**164.29**	0.96
120–140	166.65	155.43	158.45	168.38	158.41	**0.1**	**161.46**	0.95
140–160	188.43	167.21	155.82	163.72	154.90	**0**	**166.02**	0.97
160–180	179.78	167.66	162.60	154.72	160.80	**0**	**165.11**	0.97
180–200	154.56	155.68	153.80	161.58	153.82	**0**	**155.89**	0.91
200–220	152.30	159.32	143.83	152.93	155.21	**0**	**152.72**	0.90
220–400	**1154.20**	**1042.38**	**1203.75**	**1167.49**	**1212.52**	**0**	**1156.07**	6.78

### Water Distribution Characteristics of Individual Soil Layers

**FIGURE 5:**
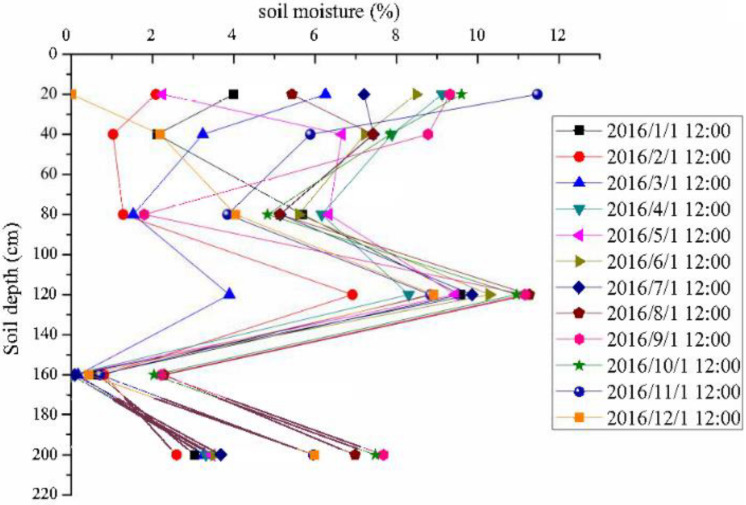
Month soil moisture changes of soil layers at different depths in 2016.

In order to study the degree of soil moisture response to precipitation in individual layers, this research chooses each layer’s soil moisture at the beginning of each month of 2016 as a representative, to observe whether the soil moisture in a specific layer is recharged. Figure 5 shows the soil moistures at depths of 20, 40, 80, 120, 160, and 200 cm at the beginning of each month. From Figure 5, the soil of PSM exhibits four distinctive layers: an evapotranspiration layer at 0–40 cm depth, a lateral root activity layer at 40–160 cm depth, a relatively dry soil layer at 160–200 cm depth, and a deep soil layer below 200 cm. The soil layer at 160–200 cm depth is usually in a relatively dry state without DSR replenishment, and it will be temporarily converted to a relatively wet state when replenishment is obtained either from heavy precipitation events or from soil moisture during the freeze-thaw process. For the 0–40 cm evapotranspiration layer, the soil moisture increases only under the effect of precipitation or snowmelt. Its moisture content decreases rapidly under the interplay of evapotranspiration and infiltration. For the 40–160 cm root activity layer, the soil moisture is recharged from infiltrated water passing through the upper layer, and it gradually decreases under the effects of infiltration and root moisture absorption. Water absorption of the PSM root system is primarily responsible for depleting the soil moisture for soils at 160–200 cm, resulting in a relatively dry state for this layer of soil. Below the 200 cm, the absorption of the PSM root system diminishes because the PSM root system can rarely penetrate a depth greater than 200 cm. Consequently, the moisture content of the soil at the 200 cm depth is higher than that at the 160 cm depth. The deep soil below 200 cm depth is of native sand soil, and Figure 5 shows that the soil moisture of this layer is recharged five times under heavy precipitation events in 2016.

### Precipitation and DSR Distribution

**FIGURE 6:**
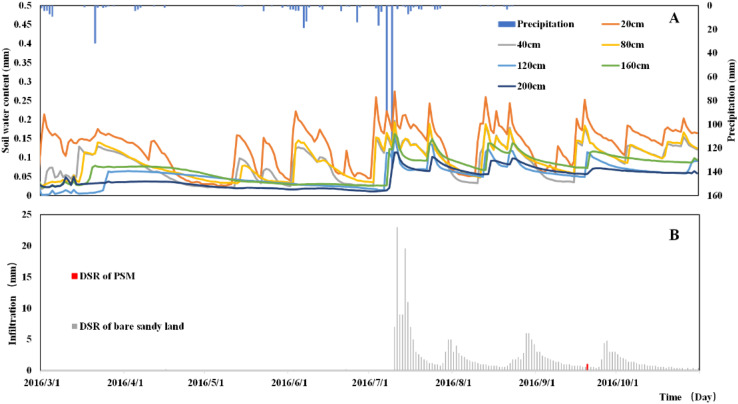
The relationship between precipitation and soil moisture distribution **(A)** and soil moisture and DSR dynamic change **(B)** in 2016.

The 40-year average precipitation in this study area is 400 mm. We define the years precipitation above this precipitation as wet years, and the years precipitation below this value as dry years. The total precipitation in 2016 is 506.4 mm, which is higher than the average annual precipitation (400 mm), thus 2016 is a wet year. As shown in Figure 6, the precipitation in 2016 is concentrated from July to August, with 86 precipitation events accounted for over the whole year in 2016. The maximum daily precipitation is 137.2 mm on July 10, and the minimum daily precipitation is 0.2 mm and it occurs multiple days in 2016. According to the distribution of soil moisture curves in each soil layer in Figure 6, the moisture content of the soil layer below 120 cm layer fluctuates during the freeze-thaw period and the summer rainy season. Before the heavy rain in July 10, no precipitation could penetrate the 120 cm depth soil layer, and the freeze-thaw water and precipitation are absorbed by the PSM root system. The results show that there are two replenishment processes of soil moisture, the freezing and thawing replenishment process of surface ice and snow deposits accumulated in winter, and the replenishment process of rainy season precipitation, the heavy precipitation has a significant effect on the replenishment of deep layer soil moisture in this region.

There is only 1 detected DSR event in the PSM plot over the entire year in 2016, on September 20, resulting in an annual DSR of 1 mm in the PSM plot in 2016. As shown in Figure 6B, all the measurable DSR events occur after September 21, and they are close to the winter freeze-thaw period, during which PSM usually ceases to grow. This implies that there are essentially no DSR events throughout the growing season of PSM in 2016. In comparison, in the bare sandy land plot, there are six measurable DSR events before the maximum precipitation event of July 10, 2016. After July 10, 2016, there are four considerable DSR events, with a total DSR amount of 281.6 mm. The distinctive DSR difference in the PSM forest plot and the bare sandy land plot shows that the PSM forest absorbs almost all the precipitation-induced infiltration in 2016, while the bare sandy land has a considerable amount of DSR at the same year, which accounts for 55.6% of the annual precipitation. This means that groundwater recharge is profoundly affected after vegetation reconstruction using PSM in the Mu Us Sandy Land, even for a wet year like 2016.

**FIGURE 7:**
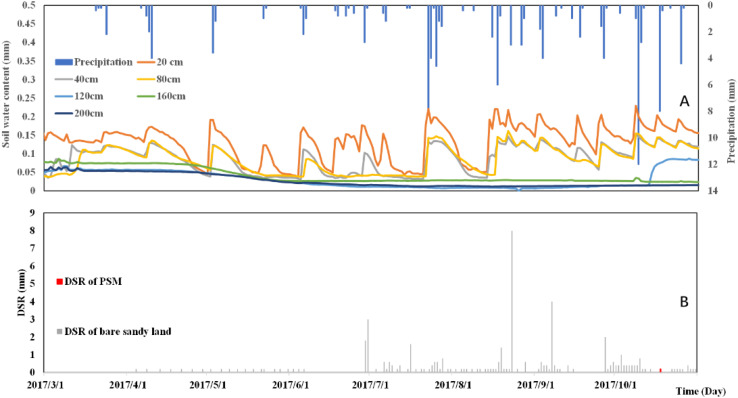
The relationship between precipitation and soil moisture distribution **(A)** and soil moisture and DSR dynamic change **(B)** in 2017.

The total precipitation in 2017 is 309 mm (less than the multi-year average precipitation of 400 mm), as shown in Figure 7, which signifies a dry year. There are 32 observed precipitation events in 2017, as shown in Figure 7. The maximum daily precipitation is 22 mm on July 22, 2017. There is a freezing period from January to March, the surface soil freezes, and the soil water content changes in each soil layers are relatively stable. March to April belongs to the freezing and thawing mixed period, frozen water in the soil layer gradually melts. Especially, the surface layer frozen water functions as a reservoir. The frozen water replenishes the soil moisture of each soil layer and the spring snowmelt recharge depth is 140 cm in 2017. April to November in 2017 is a rainy season with dynamic soil water variation. Under the interplay of precipitation-induced infiltration, soil evapotranspiration and vegetation consumption, the soil moisture fluctuates. After the rainy season, the soil begins to freeze again in December. The results show that in 2017, the total precipitation of the PSM forest land is relatively small, and the of a single precipitation is relatively small as well. Before entering the winter season, the precipitation is absorbed by the PSM root system, and no precipitation-induced infiltration can reach the 120 cm depth soil layer until later winter.

In 2017, the amount of DSR in the bare sandy land is 67.6 mm. In contrast, the amount of DSR in the PSM forest land in the same year is only 0.2 mm, as shown in Figure 7B. Since April 1, 2017, there has been a continuously recognizable DSR signal in the bare sandy land, the precipitation at this time is very small, and there should be no DSR, indicating that the previous year (2016) is a wet year, thus the soil contains a large amount of water and continues to replenish the groundwater reservoir. However, the PSM forest land has a much lower water storage due to the consumption by PSM, and the DSR below the PSM forest has been significantly reduced in 2017. As shown in Figure 6B, the soil moisture fluctuations intensely in the soil layers at 20, 40, and 80 cm depths. The soil layer from 120 to 200 cm is relatively stable, and it is only replenished by soil moisture during the freezing and thawing period (March–April). The 120 and 160 cm depth soil layers are replenished (on 14 October) due to continuous summer precipitation. The 200 cm depth soil layer remains relatively dry throughout 2017. The annual DSR underneath the PSM forest is only 0.2 mm in 2017, which is much lower than the DSR for the same plot in 2016. The lack of precipitation in 2017 causes a sharp drop in deep soil moisture infiltration.

**FIGURE 8:**
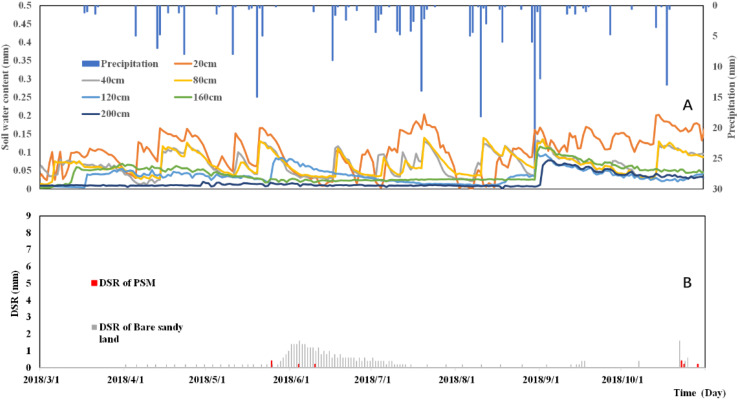
The relationship between precipitation and soil moisture distribution **(A)** and precipitation and DSR dynamic change **(B)** in 2018.

The total precipitation amount is 239.8 mm (greater than the multi-year average precipitation of 400 mm) in 2018, as shown in Figure 8, which is a dry year. There are 42 observed precipitation events throughout the year with a maximum daily precipitation of 20 mm on July 21, 2018. The precipitation distributions in 2018 and 2017 were relatively scattered, from March to October the distributions were relatively uniform, while the precipitation in 2016 was relatively concentrated in June–July. As shown in Figures 6–8, the soil moisture of the 200 cm depth soil layer in 2016 fluctuated on July 13, indicating that the precipitation recharged the 200 cm depth soil layer at this time. In 2017, the daily precipitation was generally small until there was only one rainfall event with a precipitation amount higher than 12 mm/d on October 14, and then 200 cm depth soil layer had a replenishment from precipitation. The total precipitation in 2018 was smaller than the previous 2 years, but the precipitation intensity higher than 12 mm/d event occurred many times, so the DSR of the PSM plot occurred six times, which means that the precipitation intensity is more important for the DSR of the PSM plot than the precipitation frequency, In our follow-up research, we will analyze the precipitation intensity corresponding to soil layers depth according to the signal of soil moisture fluctuation in each layer.

There is also a freezing period in January-March, in which the soil water content changes in each layer are relatively stable. The March and April belong to the freezing-thawing period. When the frozen soil water gradually melts, the soil below the surface layer is replenished by snowmelt, and the spring snowmelt recharge depth is 160 cm in 2018. Consequently, the soil moistures in various layers rise accordingly. April to November is the rainy season. Under the interplay of precipitation-induced infiltration, soil evapotranspiration and vegetation consumption, the soil moisture fluctuates greatly. The shallow soil layer begins to freeze again in December. The layers of intense soil moisture fluctuations are 20, 40, 80, 120, and 160 cm. The soil moisture change at the 200 cm depth is relatively small, and this layer is only recharged from May 14 to June 6 and on September 7, 2018.

The DSRs of the PSM forest and the bare sandy land plots are respectively 1.2 and 66.2 mm in 2018, as shown in Figure 8B. The results show that the total precipitation in 2018 exceeds the average annual precipitation. Multiple precipitations before the growing season of PSM replenish the deep soil layer moisture. The results show that in 2018, there are two precipitation replenishment processes for DSR, one is the freeze-thaw season from May to June, and the second is the precipitation process in October. The latter is caused by intensive precipitation, which replenishes the entire soil layer.

### Soil Moisture Infiltration Rate Comparison During Different Seasons

**FIGURE 9:**
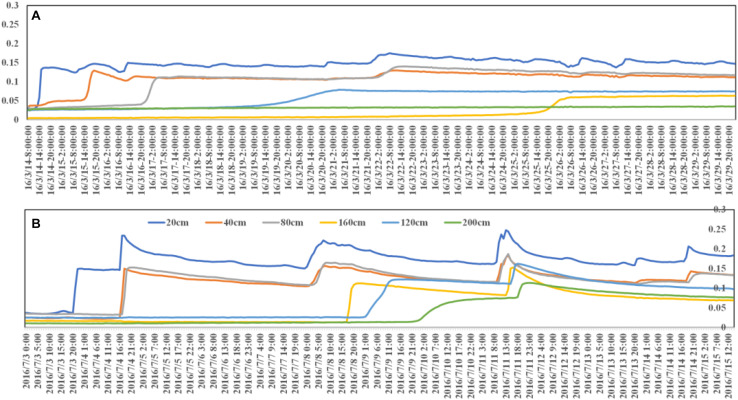
Two recharge process (snowmelt-induced and precipitation-induced) in 2016. **(A)** 20 mm/d precipitation on March 14 and **(B)** 16.6 mm/d precipitation on July 3.

The soil moisture recharge sources in the experimental area are spring snowmelt and summer precipitation. According to Figure 5, we can clearly see that the soil water recharge in different seasons varies, especially at the end of winter season and in the summer rainy season. The amount of spring precipitation in this study site is small, and snowmelt moisture is the main water source during the spring season. The germination process of vegetation or seeds in the Mu Us Sandy Land mainly depends on the water source of accumulated snowfall in winter. With the surface soil temperature increasing, the surface ice gradually melts and infiltrates to deeper soil layer. As shown in Figures 9A,B, this study chooses two typical processes for comparison: the snowmelt soil moisture recharge from February 26 to March 27, 2016, and the precipitation recharge from July 3 to 12 of 2016. During the process of snowmelt infiltration, the soil wetting front moves slowly downward, as shown in Figure 9A. It takes 2 days and 7 h for the wetting point to reach the 60 cm depth soil layer, but for the summer precipitation-induced infiltration, it takes only 1 day for the wetting front to reach the 60 cm depth soil layer.

Researches are less concerned the influence of soil temperature on the infiltration rate in modeling, and more attention is focused on the factors of soil physical properties and precipitation intensity (Dunne et al., 1991; Dunkerley, 2012). However, according to field experiments, we found that temperature had a greater influence on infiltration. In order to study the effect of temperature on the infiltration rate, we selected two precipitation events in 2016 with similar intensity (20 mm/d on March 14 and 16.6 mm/d on July 3, 2016) to study infiltration status at different seasons (with different temperatures). After two precipitation events, the soil moisture of the 20 cm depth soil layer increased rapidly, but the soil moisture changes of other depths were very different. For the precipitation event on March 14, the soil moisture of the 40 cm depth fluctuates after 1 day and 6 h after the precipitation event, the moisture of the 80 cm depth soil layer fluctuates after 2 days and 12 h, the soil moisture of the 120 cm depth soil layer fluctuates after 5 days, and the soil moisture of the 160 cm depth soil layer after 11 days then the soil moisture fluctuated, and the 200 cm depth soil layer did not receive precipitation replenishment. For the precipitation event on July 3, the soil layers at 40 cm and 80 cm depth received precipitation replenishment successively after 20 h of precipitation, the soil layers at 120 and 160 cm depth received the replenishment after 5 days, and the soil layer at 200 cm depth received replenishment after 6 days. From the entire recharge process, one can find that the precipitation intensities of the two precipitation events are almost the same, but the recharge process and the recharge depth are quite different. The recharge depth of the precipitation event on April 14 is 160 cm, the infiltration period is 11 days, and the recharge depth of precipitation on July 3 is 200 cm, and the infiltration period is 7 days. This shows that the temperature has a significant effect on the infiltration period and depth. As shown in Figures 9A,B, the *in situ* experiment inevitably encounters the superimposed effect of multiple precipitations, but the cumulative amount of the subsequent three precipitations is less than 15 mm, which has a relatively small impact on the 200 cm depth soil moisture.

These two moisture infiltration processes are shown in Figures 9A,B. There are many factors affecting the rate of precipitation-induced infiltration. A model that does not adequately consider the most relevant factors can certainly lead to erroneous simulation results. In the future, the knowledge gap between the experimental measurement results and the corresponding model simulation should be filled.

### Recharge Intensity of Different Soil Layer Infiltration

In arid and semi-arid areas, most of the precipitation will evaporate and cannot infiltrate into the soil layer and become part of the soil water, which is an important part of the available water resources for plants (Jian et al., 2015), although the latest research shows that water vapor can also be absorbed by leaves in arid and semi-arid regions (Schreel and Steppe, 2020). We define intensity of precipitation that can infiltrate into the corresponding root layer as effective precipitation. In a controlled laboratory experiment, one may calculate the precipitation infiltrating into a specific soil layer according to the soil characteristics with a proper mathematical model. In the natural environment, however, there are too many factors affecting the infiltration process, such as temperature and air humidity, wind speed, surface soil moisture, soil heterogeneity, etc. In this study, we will compute the replenishment of each soil layer by analyzing the precipitation-induced wetting point to find the minimum precipitation for infiltration to reach each individual layer, according to the results of our field test, the results are shown in Table 2.

**TABLE 2:** Precipitation produced moisture increase signal and corresponding minimum precipitation (data from 2016 to 2018).

Soil layer depths (cm)	Sum of soil moisture increase signals on each soil layer	Corresponding minimum precipitation intensity (mm/d)
20	74	2.6
40	46	3.2
80	32	3.4
120	16	8.2
160	16	10.2
200	10	13.2

During the 3-year *in situ* observation period, there were 394 observable precipitation events in total. According to the soil moisture fluctuation data recorded by the soil moisture probes, there are 294 precipitation events that have infiltrated into the soil layer below 200 cm depth.

Infiltration results in elevated soil moisture. Each time when infiltration reaches a designated depth, it leaves a crest signal of soil moisture. Based on the comparison between the time of crest signals and the time of precipitations, the minimum precipitation amount can be determined by the crest signals at different soil depths. Antecedent soil moisture conditions also affect infiltration depth, shallow soil moisture in the semi-arid sandy land will soon return to a relatively dry state because of evapotranspiration. This research describes the general state of soil moisture fluctuations in the experimental area after precipitation. Statistics of precipitation data from 2016 to 2018 and fluctuations in soil moisture in each layer are shown in Table 2, which demonstrates that for infiltration to reach the soil layers at depths of 20, 40, 80, 120, 160, and 200 cm, the required daily minimum precipitation intensities are 2.6, 3.2, 3.4, 8.2, 8.2, and 13.2 mm, respectively. Infiltration depth and precipitation are not linearly related. This suggests that in the Mu Us Sandy Land, infiltration will cease to exist when the daily precipitation is less than 2.6 mm, and DSR may be detected only when the daily precipitation becomes greater than 13.2 mm. However, according to the 3-year *in situ* experimental record, one can see that for some precipitation events whose daily intensities are greater than 13.2 mm, there are no corresponding DSR events detected. Such evidence shows that the precipitation is not the only factor controlling DSR. Besides the precipitation intensity, other factors may also be relevant. Further research is needed to understand those factors impacting the soil moisture dynamics and DSR. One should note that the above infiltration depth is not based on mathematical calculations, but on the basis of the soil moisture fluctuation data monitored by the soil moisture probe at the experimental site.

### Redistribution of Precipitation in the PSM Forest

Figure 5 shows that during the seasonal frozen-soil period, soil moisture is relatively stable. The monthly average values for soil moistures of different soil layers in January and December of 2016 are used as the start and end soil moisture values. Although the precipitation amount varies from 2016 to 2018, other environmental factors in this area are basically the same, and soil moistures are similar. To figure out the PSM water balance from 2016 to 2018, one has:

(2) P-DSR-⁢ET=∂⁡W

where P is precipitation, ET is evapotranspiration, and δW is the whole 200 cm soil layer moisture change. Runoff is not included in above water balance equation because it does not occur during the experiment. As precipitation, DSR, and δW can be accurately measured, ET can be calculated by above equation.

Table 3 shows the precipitation, DSR, δW, and the computed ET for 2016–2018. Based on Table 2, one can see that precipitation has played a role in regulating and replenishing soil moisture for both shallow and deep soil layers. For the shallow soil layer, evapotranspiration in the dry year (like 2018) consumes stored water from previous wet year (2016), but in the wet year (like 2016), precipitation recharges the shallow soil layer. The recorded DSR values in 2016–2018 are very small, as compared to other terms in above Eq. 2, indicating that under the existing vegetation cover and rainfed conditions, the precipitation is barely able to support the shallow groundwater ecosystems, and has almost no capacity to provide recharge for groundwater reservoir in the region. However, in the bare sandy plot, the precipitation indeed can provide moderate recharge for groundwater reservoir, as reflected in the sizable annual DSR values there. In semiarid regions such as the Mu Us Sandy Land, precipitation varies considerably every year, and the year of 2016 may not be representative of the long-term average behavior of DSR in this region as the precipitation of this year is higher than the average annual precipitation of 400 mm. One can see that in different years, vegetation water consumption patterns are different, and evapotranspiration decreases in dry year and increases in wet year. One common feature among wet and dry years is that precipitation-induced infiltrated water is trapped in shallow soil layers and then consumed by PSM. To understand the long-term behavior of DSR and soil moisture dynamics in the semiarid regions such as the Mu Us Sandy Land, one must carry out a multi-year (preferably a decade long) experiment.

**TABLE 3:** Water distribution of the rainfed PSM and bare sandy plot.

Year	Plot	Precipitation (mm)	DSR (mm)	δW (mm)	ET (mm)
2016	PSM	506.4	1	38.06	466.94
	BSL	506.4	273.6	95.67	137.13
2017	PSM	309	0.4	−16.00	324.60
	BSL	309	67.7	61.89	179.41
2018	PSM	239.8	1.2	54.75	183.85
	BSL	239.8	55.2	96.8	87.8

As shown in Table 3, comparing the PSM plot and the bare soil land (BSL) plot, it can be found that the ET is 466.94 mm and the DSR is 1 mm for the PSM plot in 2016; while the ET is 137.13 mm and the DSR is 237.6 mm for the BSL plot in 2016. From this observation one can conclude that due to vegetation reconstruction, the DSR is significantly reduced and the ET is significantly increased in this year. 2018 was a relatively dry year with an annual precipitation of 239.8 mm. Due to the decrease in precipitation in 2018, the DSR has decreased in both plots in this year. The DSR in the PSM plot is 1.2 mm and the BSL plot DSR is 55.2 mm in 2018, which are significantly lower than their counterparts in 2016.

In the 3-year period of 2016–2018, DSR has always existed, indicating that precipitation can replenish deep groundwater in this region. However, compared with bare sandy land, the amount of replenishment in PSM plot has dropped sharply, indicating that vegetation reconstruction will inevitably change the results of water redistribution in this region. Studies have shown that precipitation in the 3NSP region has also increased, which is also due to increased evapotranspiration due to the presence of vegetation (Meng et al., 2020). The amount of evapotranspiration of PSM in this study is different in every year, which is consistent with the results of previous studies, PSM can survey in dry years by reducing transpiration and storing water in the stems in dry years (Cheng et al., 2021).

### Insights Gained From This Study

In semi-arid areas, the main limiting factor for ecosystems is available water resources (Skarpe, 1991; Gao et al., 2014). Therefore, the key to understand the vegetation ecosystem in semi-arid areas is to study the supply of water resources (Cheng et al., 2017; Cheng et al., 2018). The PSM has been in existence in the study area for more than 30 years, so the purpose of this study is to find out whether there is sufficient water resource available in the region to support vegetation ecosystem, through the measurement of deep soil water recharge (or DSR). The “sustainable” growth of plants in this study means that water resource from precipitation can meet the growth needs of PSM, and still have excess amount of water to replenish deep soil layer (to recharge the deep groundwater system beyond the root zones of plants). In this study, the soil moisture distribution of PSM is studied using a newly designed lysimeter to see whether the soil layer below the root layer could produce DSR or not.

Lysimeter has been developed for hundreds of years and is widely used in the field of water balance research (Boast and Robertson, 1982; Liu et al., 2002; López-Urrea et al., 2006). The conventional lysimeter has a size limitation, and is ineffective in measuring trees and other large plants (Fritschen et al., 1977). The new lysimeter solves the shortcomings of conventional instruments that cannot be observed *in situ* due to the high cost (Cheng et al., 2017; Cheng et al., 2018). Conventional lysimeter need to excavate soil and transplant trees. Due to the large root system, conventional instruments cannot observe tall trees. This study uses a newly designed lysimeter to measure the deep soil moisture infiltration for water balance investigation (Cheng et al., 2017). From the field observation results of 2016–2018, In Mu Us Sandy Land, there is no direct relationship between annual precipitation and the amount of penetration or DSR, DSR is related to heavy precipitation, which can be inferred that it is not feasible to use an infiltration coefficient to estimate the DSR. In semi-arid region, DSR is closely related to vegetation coverage, precipitation patterns and other factors.

Vegetation depends on precipitation, and roots are concentrated in shallow soil layers. Measuring the water absorption of topsoil and interception of the canopy are complicated processes and often involves a great degree of uncertainty. This research, however, regards the canopy and topsoil as an integrated entity. And by measuring the amount of water entering the entity (precipitation) and the amount of water leaving the entity (DSR), one may conduct a water balance computation to calculate the amount of overall evapotranspiration.

### Limitations and Future Works

This study monitors the distribution of soil moisture of PSM forest under the rainfed conditions in a 3-year period (2016–2018), and try to understand whether the soil moisture is sufficient (the generation of DSR on 200 cm depth soil layer indicates that the precipitation meets the growth of PSM and excess water replenishes the deep soil) under existing precipitation conditions. One should be cautious that the 3-year (2016–2018) soil moisture measurements presented in this study may not be representative of the long-term ecohydrological process in the region. Therefore, it is necessary to perform long term (such as decades long) observation and analysis in the region to see if the studied species (PSM) can develop sustainably or not over decades as some artificial trees may have life cycles over decades long. Another notable point is that the adaptability of long-lived woody species may not be based solely on water, temperature, light, and soil texture. Despite such limitations, this 3-year investigation offers an important step for understanding the soil moisture dynamics of sand-fixing PSM forest in a semi-arid region. Fortunately, these 3 years (2016–2018) happen to encompass rather dramatically different weather patterns in the region (wet versus dry years), thus this study offers insights on the function of the PSM forest under both wet and dry years.

This research mainly focusses on the movement of liquid water (DSR) under shallow soil layer (such as 2 m depth) with groundwater levels as low as 8 m in Mu Us Sandy Land. How important is the transportation of water vapor at the research site is an open question that should be answered in future (Zeng Y. et al., 2009). It is speculated that even for groundwater with a depth of several 100 m, due to thermal gradients, soil matrix potential, or diffusion and dispersion processes, vapor migration may continue, specially in a desert area (such as this location), and is an important water source for desert ecosystem (Zeng et al., 2011). In the past few decades, relevant studies on Chinese deserts have shown that water vapor transport plays an important role in regulating infiltration and land surface evaporation (Scanlon and Milly, 1994). Further studies are needed to quantify the importance of vapor flow and condensation at this study site.

## Conclusion

The following conclusions can be drawn from this study:

1. Even though the precipitation in the Mu Us Sandy Land fluctuates over the 3-year period investigated in this study, in general the precipitation recharge is greater than the demand of PSM and extra recharge can pass through the PSM root zone to recharge the groundwater.
2. Comparing bare sandy land and PSM forest land, we can find that the fine roots of PSM in the Mu Us Sandy Land are distributed in the shallow soil layer of 0–100 cm depth, and the precipitation-induced infiltration is intercepted and stored in shallow soil layers as well.
3. Due to the PSM, precipitation is intercepted by roots layer in the shallow soil, and the amount of DSR decreased from 273.6 mm in bare sand to 1 mm (wet year, 2016), and from 55.2 to 1.2 mm (dry year, 2018).
4. For precipitation-induced infiltration to reach depths of 20, 40, 80, 120, 160, and 200 cm soil layers, the daily precipitation intensities have to be greater than 2.6, 3.2, 3.4, 8.2, 8.2, and 13.2 mm, respectively.
5. In dry and wet years, the evapotranspiration of PSM forest land ranges from 183.85 to 466.94 mm, and PSM has demonstrated good drought tolerance capacity.

## Data Availability Statement

The raw data supporting the conclusions of this article will be made available by the authors, without undue reservation.

## Author Contributions

YBC and HBZ conceived the idea. YBC, QOJ, XLL, and YQW conducted the analyses. YBC, MCS, XLL, WBY, and ZMX provided the data. All authors contributed to the writing and revisions.

## Conflict of Interest

The authors declare that the research was conducted in the absence of any commercial or financial relationships that could be construed as a potential conflict of interest.

## Abbreviations

3NSP: Three North Shelterbelt Program
PSM: Pinus sylvestris var. mongolica
DSR: deep soil recharge
ET: evapotranspiration.
